# Multi-tissue transcriptome analysis of two *Begonia* species reveals dynamic patterns of evolution in the chalcone synthase gene family

**DOI:** 10.1038/s41598-021-96854-y

**Published:** 2021-09-07

**Authors:** Katie Emelianova, Andrea Martínez Martínez, Lucia Campos-Dominguez, Catherine Kidner

**Affiliations:** 1 Royal Botanic Gardens Edinburgh, 20a Inverleith Row, Edinburgh, EH3 5LR UK; 2 Dementia Research Institute at the University of Edinburgh, Edinburgh, UK; 3 School of Biological Sciences, University of Edinburgh, King’s Buildings, Mayfield Rd, Edinburgh, EH9 3JU UK

**Keywords:** Evolution, Phylogenetics, Natural variation in plants, Plant evolution

## Abstract

*Begonia* is an important horticultural plant group, as well as one of the most speciose Angiosperm genera, with over 2000 described species. Genus wide studies of genome size have shown that *Begonia* has a highly variable genome size, and analysis of paralog pairs has previously suggested that *Begonia* underwent a whole genome duplication. We address the contribution of gene duplication to the generation of diversity in *Begonia* using a multi-tissue RNA-seq approach. We chose to focus on chalcone synthase (CHS), a gene family having been shown to be involved in biotic and abiotic stress responses in other plant species, in particular its importance in maximising the use of variable light levels in tropical plants. We used RNA-seq to sample six tissues across two closely related but ecologically and morphologically divergent species, *Begonia conchifolia* and *B. plebeja*, yielding 17,012 and 19,969 annotated unigenes respectively. We identified the chalcone synthase gene family members in our *Begonia* study species, as well as in *Hillebrandia sandwicensis*, the monotypic sister genus to *Begonia*, *Cucumis sativus*, *Arabidopsis thaliana*, and *Zea mays*. Phylogenetic analysis suggested the CHS gene family has high duplicate turnover, all members of CHS identified in *Begonia* arising recently, after the divergence of *Begonia* and *Cucumis*. Expression profiles were similar within orthologous pairs, but we saw high inter-ortholog expression variation. Sequence analysis showed relaxed selective constraints on some ortholog pairs, with substitutions at conserved sites. Evidence of pseudogenisation and species specific duplication indicate that lineage specific differences are already beginning to accumulate since the divergence of our study species. We conclude that there is evidence for a role of gene duplication in generating diversity through sequence and expression divergence in *Begonia*.

## Introduction

*Begonia* is one of the most diverse Angiosperm genera, with more than 2000 species described to date^1^. The genus is thought to have originated in Africa between 24 and 45 MYA and since then diversified across South America and Asia^2^, where it occupies a wide range of niches, facilitated by a diversity of vegetative forms across species^3^.

Strong population structure, high levels of drift, and genetic divergence at local scales are thought to contribute to the high species diversity in *Begonia*^4^. Endemism is very common^5^, and strong population structure is known to coincide with high variation in morphological characteristics such as leaf shape and size^6^.

**Figure 1:**
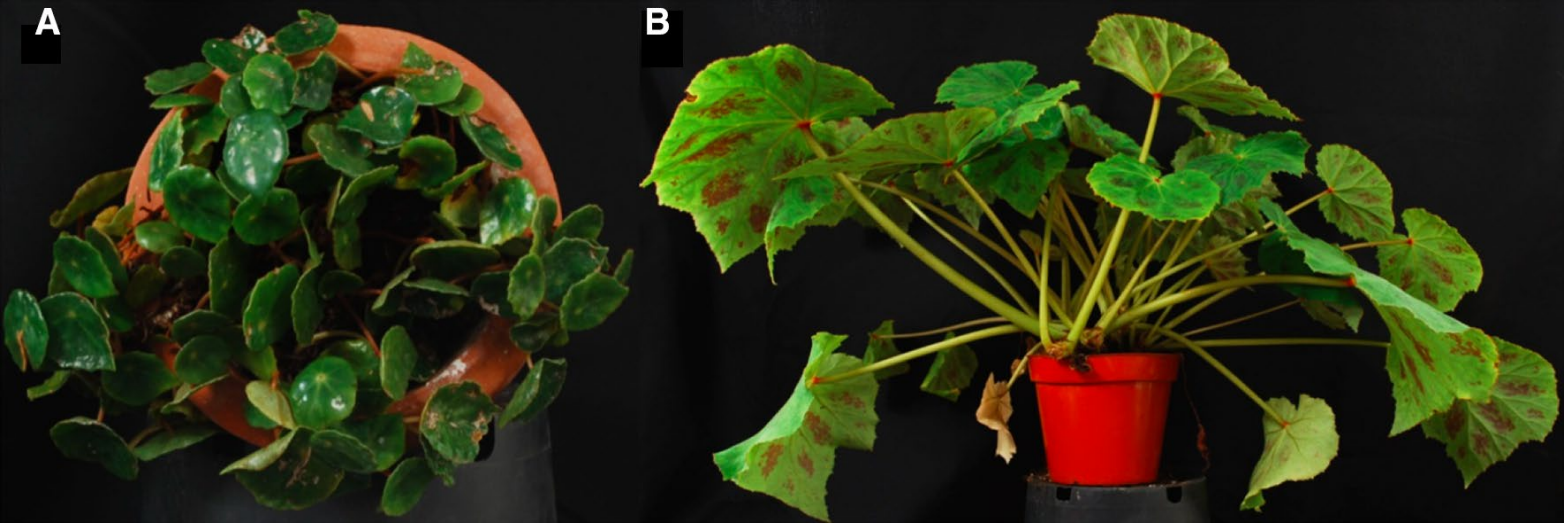
Photographs of study species used. *B. conchifolia* (**A**) and *B. plebeja* (**B**).

*Begonia* has also been shown to have highly variable genome sizes^7^, and evidence of whole genome duplications has been identified from paralog kS peaks^8^. The contribution of gene and genome duplication has long been associated with the evolution of phenotypic novelty^9^, and the effect of duplication on morphological diversity in *Begonia* is ongoing. This study used multi tissue RNA-seq to study diversification in duplicated genes in two closely related but morphologically divergent species *B. conchifolia* and *B. plebeja* (Fig. 1).

*Begonia conchifolia* is a small terrestrial plant with long-lived fleshy peltate leaves and small white flowers. It has a restricted distribution in wet rainforests across southern Mexico and Central America. *Begonia plebeja,* which also has a terrestrial growth form, is more widespread, occupying seasonally dry forests in northern Mexico. It has larger, thinner leaves which are deciduous in some populations and often blotched. Flowers are larger and sometimes tinged with pink^10^.

The recent divergence of *B. conchifolia* and *B. plebeja* and the distinct ecological niches they now occupy provides a good model to study changes in duplicate gene sequence and expression post speciation. The different environments inhabited by the two study species suggest they may face different ecological challenges; the open and dry habitat of *B. plebeja* compared to the darker, shaded understorey habitat of *B. conchifolia* have wide ranging consequences, including access to light, water availability, and risk of pathogen and fungal attack. Here we use multi-tissue RNA-seq to interrogate patterns of duplicate gene evolution at the sequence and expression level, focussing on evolutionary and duplication patterns of the anthocyanin biosynthetic gene chalcone synthase (CHS, EC 2.3.1.47).

CHS is the first committed step of the anthocyanin biosynthesis pathway^11^, and is a crucial enzyme in the production of compounds used in biotic and abiotic stress responses^12–14^. Anthocyanin pigments are important in the attenuation of high UV exposure, preventing PSII inhibition and the reduction of carbon intake^15,16^. Distribution of anthocyanins across a variety of tissues helps low light dwelling plants make the most use of intermittent high intensity sunflecks^17,18^ while avoiding photodamage and attenuating stress response through ROS scavenging^19^.

Uncovering the genetic and genomic basis upon which phenotypic and biochemical changes occurred may shed light on the mechanism of divergence in *B. conchifolia* and *B. plebeja*, and may answer wider questions about diversification in the genus *Begonia*. The wide ranging role CHS plays in biotic and abiotic responses makes it a good initial candidate for investigating patterns of diversification in sequence and expression pattern. With our investigation of CHS, we hope to shed light on the evolution of a historically dynamic^20,21^ gene family pre- and post-speciation of *B. conchifolia* and *B. plebeja*.

## Methods

### Tissue sampling and RNA extraction

Plant tissue was donated from plants in the living collection at Royal Botanic Gardens Edinburgh. The tissues chosen for study, mature leaf, mature petiole, vegetative bud, female flower, male flower and root, were harvested between 9 and 10am between January and May 2015 from *B. conchifolia* (Accession Number: 20042082) and *B. plebeja* (Accession Number: 20051406) (evergreen genotype). Both accessions were housed in the same greenhouse and grown under the same conditions. Leaves were the first fully expanded leaf on the axis, petioles were from these leaves, flowers were staged between tepals just opening and tepals fully expanded, roots were young white roots within 5–10 cm of the apex. RNA from three biological replicates per tissue was extracted using the phenol chloroform protocol^22^ and quantitated using Qubit (Thermo Fisher). Sample purity was estimated using a NanoVue Spectrophotometer.

### Sequencing

Library preparation and sequencing, carried out by Edinburgh Genomics, consisted of preparation of TruSeq mRNA-seq libraries, and generation of c. 240 million 150 base pair paired-end reads on one lane of a HiSeq rapid v1 machine. Raw reads are stored in the European Nucleotide Archive under the study accession PRJEB26711.

### Removal of contaminants from RNA-seq reads

BlobTools^23^ was used to screen for and remove contaminants from assemblies. Reads were first adapter trimmed using Trimmomatic^24^ using a 4 base sliding window and a minimum mean quality of 15. Leading and trailing bases lower than quality score 3 were trimmed. Total adapter trimmed reads were assembled using Trinity v2.5.1^25^ using default parameters. Coverage was estimated by mapping reads back to their corresponding species assembly with STAR v2.5.3a^26^ using default parameters. Finally, contigs from each assembly were used as a query to search against the NCBI nucleotide database (nt) with BLAST v2.2.28 for taxonomy assignment.

Using the assembly, taxonomy and coverage files, BlobPlots were created to visualise contigs partitioned by taxon, GC content, and coverage. Contigs which were annotated as Streptophyta were used to extract associated reads belonging to this taxon using the BlobTools bamfilter functionality.

### Assembly and quality control

Decontaminated total reads were assembled using Trinity v2.6.4 using default parameters. The longest isoform per gene was obtained with Trinity utility scripts to obtain a set of unigenes for each assembly.

Transcriptome assembly quality was assessed with Transrate v.1.0.3^27^. Transrate reports basic metrics for a transcriptome assembly and provides quality information for assembled contigs using coverage and accuracy information by mapping reads to assembled contigs.

Transcriptome assembly completeness was estimated using BUSCO v4.0.0^28^, using transcriptome mode.

### Annotation

The Trinotate v3.2.1 pipeline^29^ was used to functionally annotate unigenes for each species. Unigenes were searched against the Swissprot database with blastx v2.2.28 using an E value cutoff of 1e^−3^ and setting maximum target sequences to 1.

Most likely longest ORF peptide sequences were predicted from unigenes with Transdecoder v5.5.0. The resulting predicted peptides were used to search against the Swissprot database with blastp v2.2.28, using an E-value cutoff of 1e^−3^ and setting maximum target sequences to 1. Protein domains were identified by searching predicted longest ORF peptides against the Pfam database using hmmscan v3.1b1. Blast homologies from blastp and blastx results and Pfam domains from hmmscan results were loaded into the Trinotate provided SQLite database, and an annotation report was generated.

### Coverage

Decontaminated reads per tissue and replicate were mapped to unigenes for each species with STAR v2.5.3a using default parameters. Read counts were summarized across features using Subread’s FeatureCounts v1.5.2^30^, not including read pairs which map to different contigs.

### Expression normalization

EdgeR^31^ was used to normalise counts generated by FeatureCounts. Library size and composition was accounted for using TMM (trimmed mean of M-values) normalisation, and average FPKM values were calculated for replicates of tissue groups per species.

### Characterization of CHS

The *Arabidopsis thaliana* protein sequence (AT5G13930) was used to search the *B. conchifolia* and *B. plebeja* nucleotide databases of longest assembled isoforms with tblastn, using a conservative estimate of homology^32^ of E value threshold of 1e^−20^ and a percent identity threshold of 50%.

The same strategy was used to find homologs of CHS in the draft *Hillebrandia sandwicensis* genome^33^, tblastn coordinates were used to extract CHS coding sequences from contigs containing hits. Nucleotide sequences of positive hits in *B. conchifolia*, *B. plebeja* and *H. sandwicensis* were aligned with the *A. thaliana* CHS cDNA sequence and a *Zea mays* homolog of CHS (C2, gene symbol LOC100274415) with Geneious^34^ using the Geneious aligner, specifying global alignment with free end gaps, a similarity threshold of 65%, and a cost matrix of 5/− 4 for matches and mismatches respectively.

### Phylogenetic analysis of CHS

Sequences with an overlap shorter than 200 bp with all other sequences were not included in further analysis, as per previous studies^35^. Intronic sequence introduced by genomic sequences was excised, and conserved sequence composed of the first and second exon was extracted. The alignment was manually checked and corrected prior to further analysis.

The final alignment of CHS sequences was used to perform a model selection procedure using Model Generator^36^ based on the Akaike Information Criterion (AIC).

RAxML^37^ was used to construct a gene tree using the GTR + R substitution model with 1000 bootstrap replicates.

### Analysis of conserved sites in CHS

The peptide sequence of all *Begonia* CHS sequences was identified by translating sequences into all six reading frames and aligning with the *A. thaliana* CHS (AT5G13930) using the Geneious aligner with default settings, identifying the correct reading frame of *Begonia* CHS by greater than 50% sequence similarity to the *A. thaliana* protein sequence. Previously identified conserved sites^11,38^ were mapped onto the alignment of correctly translated *Begonia* peptide sequences and the *A. thaliana* protein sequence.

### Selection analysis of CHS

Codeml from the PAML package of programs^39^ was used to estimate the rate of nonsynonymous substitutions to synonymous substitutions (dN/dS). First, we used the peptide alignment of *Begonia* CHS to create a translational alignment of the nucleotide coding sequences of each pair of orthologous transcripts, where a pair was available, using the Geneious aligner with default settings. A pairwise analysis of dN/dS was performed on each orthologous pair of *Begonia* CHS sequences using codeml, setting runmode to − 2, running on codon data, and specifying one dN/dS ratio to be calculated for the whole alignment.

### Statement on plant guidelines

Collection of plant material complies with relevant institutional, national, and/or international guidelines and legislation.

## Results

To remove sequences sampled from other taxa during RNA extraction, BlobTools was used to classify assembled transcript sequences, and only reads contributing to sequences classified as Streptophyta were used for all downstream analysis.

BlobTools infers taxonomic annotation from a similarity search of the input sequences against a public sequence collection (e.g. NCBI nt), and determines the taxonomy of each sequence using a taxrule algorithm. Coverage and GC content of the annotated sequences are plotted in order to visualise the partitioned sequences and perform downstream contaminant screening.

Screening for contaminants revealed the majority of taxonomically assigned transcripts belonged to Streptophyta, with 62,082 and 68,696 transcripts from *B. conchifolia* and *B. plebeja* respectively assigned to the taxon (supplementary Figs. 1 and 2). The next most frequent taxon represented by annotated transcripts in *B. conchifolia* is Arthropoda with 3629 transcripts, and Ascomycota in *B. plebeja* with 7309 transcripts, representing plausible sources of contamination from a greenhouse setting.

The sequence length weighted span of coverage in both species’ BlobPlots shows Streptophyta having the second highest peak of coverage after no-hit sequences, and the widest span, reflecting the range of expression levels of the transcripts screened.

Using the BlobPlot information, reads from both annotated contaminant and no-hit transcripts were removed from further analysis totalling around 80 million reads in *B. conchifolia* and 64 million in *B. plebeja* (supplementary table 1).

**Figure 2:**
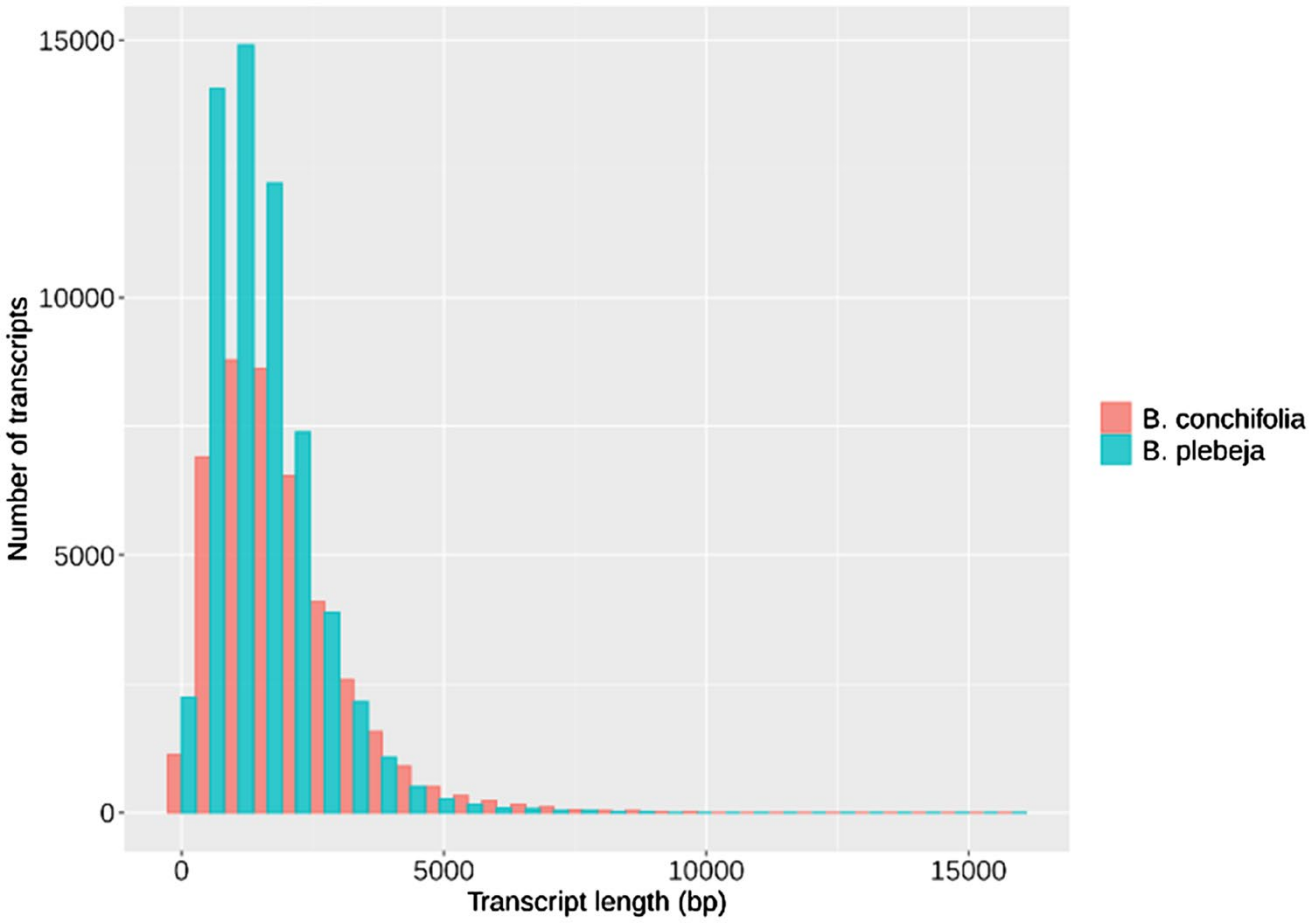
Sequence length histogram showing *B. conchifolia* and *B. plebeja* sequence length distribution.

Comparison of sequence length distributions showed that *B. plebeja* assembled transcripts were skewed towards shorter transcripts, while *B. conchifolia* transcripts had fewer shorter transcripts and an increasingly greater number of longer transcripts (Fig. 2). *Begonia conchifolia* had higher N50 and N90 (2381 and 1090) compared to *B. plebeja* (1905 and 842) (Table 1), and BUSCO assessment of transcriptome completeness (Table 2) showed that *B. plebeja* had marginally poorer scores for transcript completeness and fragmentation, however both transcriptomes showed over 80% completeness.

**Table 1:** Assembly statistics for *B. conchifolia* and *B. plebeja* before and after contaminant removal.

	*B. conchifolia*	*B. plebeja*
Number of seqs	42,614	59,106
Number of unigenes	17,012	19,969
Smallest seq	201	201
Largest seq	15,923	16,037
N over 1 kb	31,876	37,865
N over 10 kb	28	16
N with ORF	32,848	43,119
N90	1090	842
N50	2381	1905

**Table 2:** BUSCO assessment of transcriptome completeness for *B. conchifolia* and *B. plebeja*.

BUSCO category	*B. conchifolia*	*B. plebeja*
Complete	362 (85.2%)	351 (82.5%)
Complete single copy	352 (82.8%)	344 (80.9%)
Complete duplicated	10 (2.4%)	7 (1.6%)
Fragmented	23 (5.4%)	36 (8.5%)
Missing	40 (9.4%)	38 (9%)
Total	425	425

**Figure 3:**
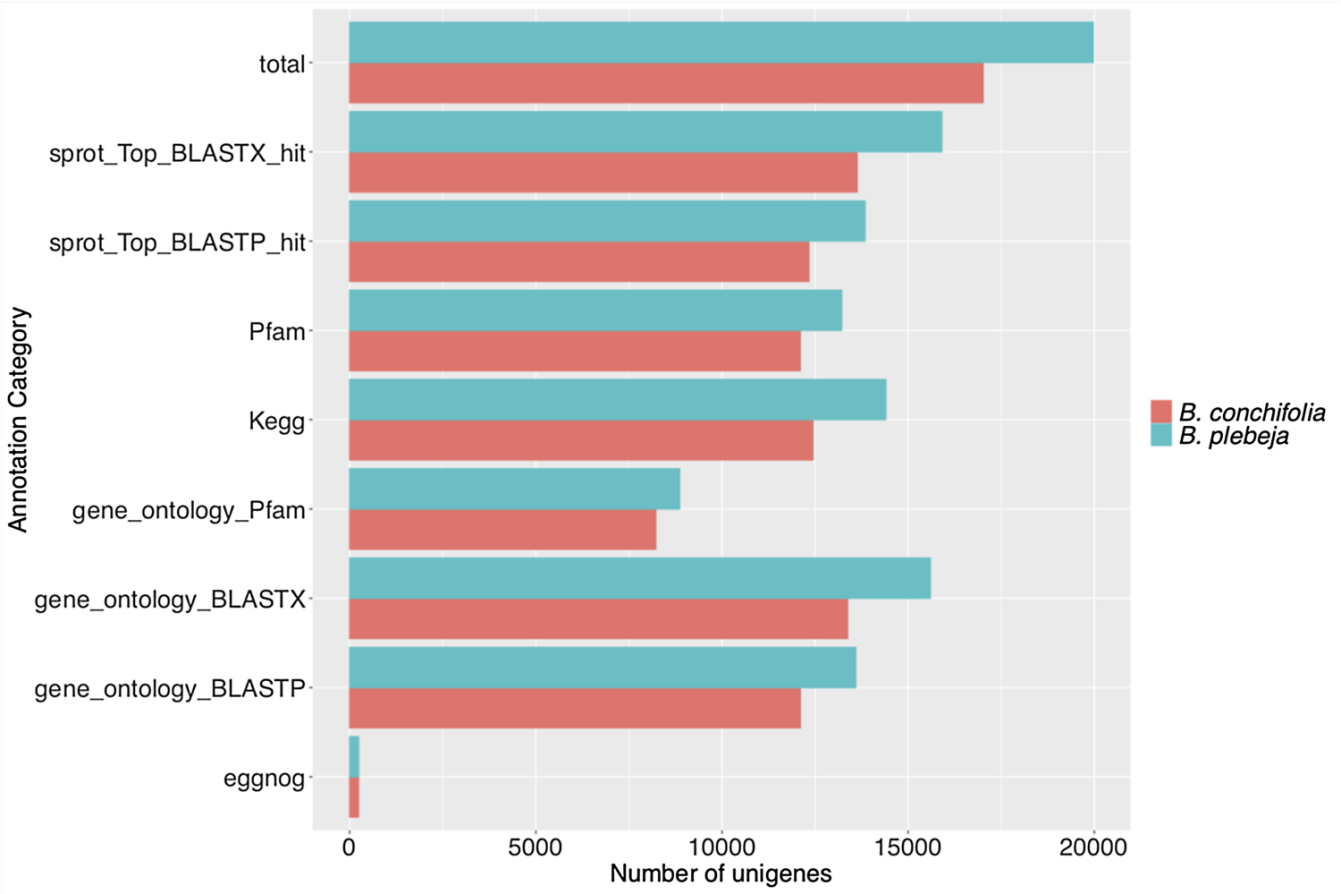
Number of unigenes annotated using different annotation categories in *B. conchifolia* and *B. plebeja*.

The Trinotate pipeline was used to annotate 17,012 *B. conchifolia* and 19,969 *B. plebeja* unigenes (supplementary files 3 and 4). More unigenes were annotated in *B. plebeja* than in *B. conchifolia* within each annotation source (Fig. 3), likely due to the larger number of input transcripts. Concordantly, *B. plebeja* also has more transcripts without any annotation, 16.65% and 17.48% of unigenes are unannotated in *B. conchifolia* and *B. plebeja* respectively. Roughly equal proportions of unigenes from both species were annotated to Streptophyta (70.87% in *B. conchifolia* and 70.51% in *B. plebeja*).

Unigenes which shared sources of annotation were identified using UpSet plots, showing that, due to very sparse EggNOG annotation (Fig. 3), the largest group of unigenes sharing annotation sources were annotated with all sources except for EggNOG (supplementary Figs. 5 and 6), *B. conchifolia* having 7217 unigenes in this group and *B. plebeja* 7787.

Unigene presence or absence across tissues was compared for *B. conchifolia* and *B. plebeja*, using a cutoff of 1 FPKM for whether a transcript is present or absent in a tissue. UpSet plots were used to visualise the shared and tissue specific distribution of unigene expression (supplementary Figs. 3 and 4). The majority of unigenes are expressed in all tissues (11,495 unigenes in *B. conchifolia* and 13,609 unigenes in *B. plebeja*).

Both species had a high frequency of unigenes expressed uniquely in root (528 and 502 unigenes in *B. conchifolia* and *B. plebeja* respectively) and in male flower (223 and 359 unigenes in *B. conchifolia* and *B. plebeja* respectively).

To identify unigenes which are uniquely expressed in each tissue, we used a 1 FPKM cut-off for unigene expression, identifying unigenes which are expressed in a single tissue and not expressed in any other tissues. For example, a unigene which is uniquely expressed in root has an expression of more than 1 FPKM in root, and less than 1 FPKM in all other tissues. To identify the functional categories of genes uniquely expressed per tissue, we mapped GO terms to tissue specific unigenes (plotted in supplementary Figs. 7and 8). The total number of GO terms mapped to these uniquely expressed unigenes, here referred to as unique GO terms (UGT), is shown in Table 3. *Begonia conchifolia* and *B. plebeja* had a comparable number of UGTs across tissues. Both species had fewer UGTs in female flower compared to male flower (199 and 131 in female flower and 772 and 924 in male flower in *B. conchifolia* and *B. plebeja* respectively). Of all tissues, leaf had the least UGTs in both species (69 and 0 in leaf in *B. conchifolia* and *B. plebeja* respectively), and root tissue had the greatest number (1183 and 1820 in leaf in *B. conchifolia* and *B. plebeja* respectively). Tissues which had the biggest difference in number of UGTs between species were petiole (a difference of 225 UGTs) and vegetative bud (a difference of 341 UGTs).

**Table 3:** Number of GO terms mapped to unigenes expressed uniquely in each tissue in *B. conchifolia* and *B. plebeja*.

Tissue	*B. conchifolia*	*B. plebeja*
Female flower	199	131
Leaf	69	0
Male flower	772	924
Petiole	338	113
Root	1183	1820
Vegetative bud	102	443

After alignment of the CHS copies identified, any CHS sequences which had an overlap of less than 200 bp within the conserved coding sequence with any other sequence were discarded (supplementary file 1). A maximum likelihood tree was inferred from the remaining subset of CHS copies identified in *B. conchifolia* and *B. plebeja*, using *Z. mays* as an outgroup, and including the CHS sequence of *Cucumis sativus,* the closest relative to *Begonia* with a publicly available genome sequence^40^, and *H. sandiwcensis*, the monotypic sister genus to *Begonia* (supplementary file 2). Four copies of CHS were identified in *B. conchifolia* and five copies in *B. plebeja.*

At least one more copy exists in both species (supplementary table 2), but due to incomplete sequence reconstruction, it is not possible to assign a copy number with any certainty. Of the nine CHS sequences included for *B. conchifolia* and *B. plebeja*, four pairs of closely related orthologs were identified in *B. conchifolia* and *B. plebeja*, as well as one additional *B. plebeja* duplicate.

The phylogenetic tree reconstructed for CHS copies from *Begonia*, *Hillebrandia*, *C. sativus, A. thaliana* and *Z. mays* revealed duplicates from *B. conchifolia* and *B. plebeja* arose after the divergence of *Begonia* and its closest sequenced neighbour in the Curcurbitales, *C. sativus*. *Begonia* sequences were obtained from RNA-seq, therefore it is not possible to confirm that no older duplicates exist in *B. conchifolia and B. plebeja,* however the absence of older duplicates in the genome of *H. sandiwcensis,* the monotypic species of *Begonia’*s sister genus *Hillebrandia* supports a pattern of high duplicate turnover in the *Begoniaceae*.

**Figure 4:**
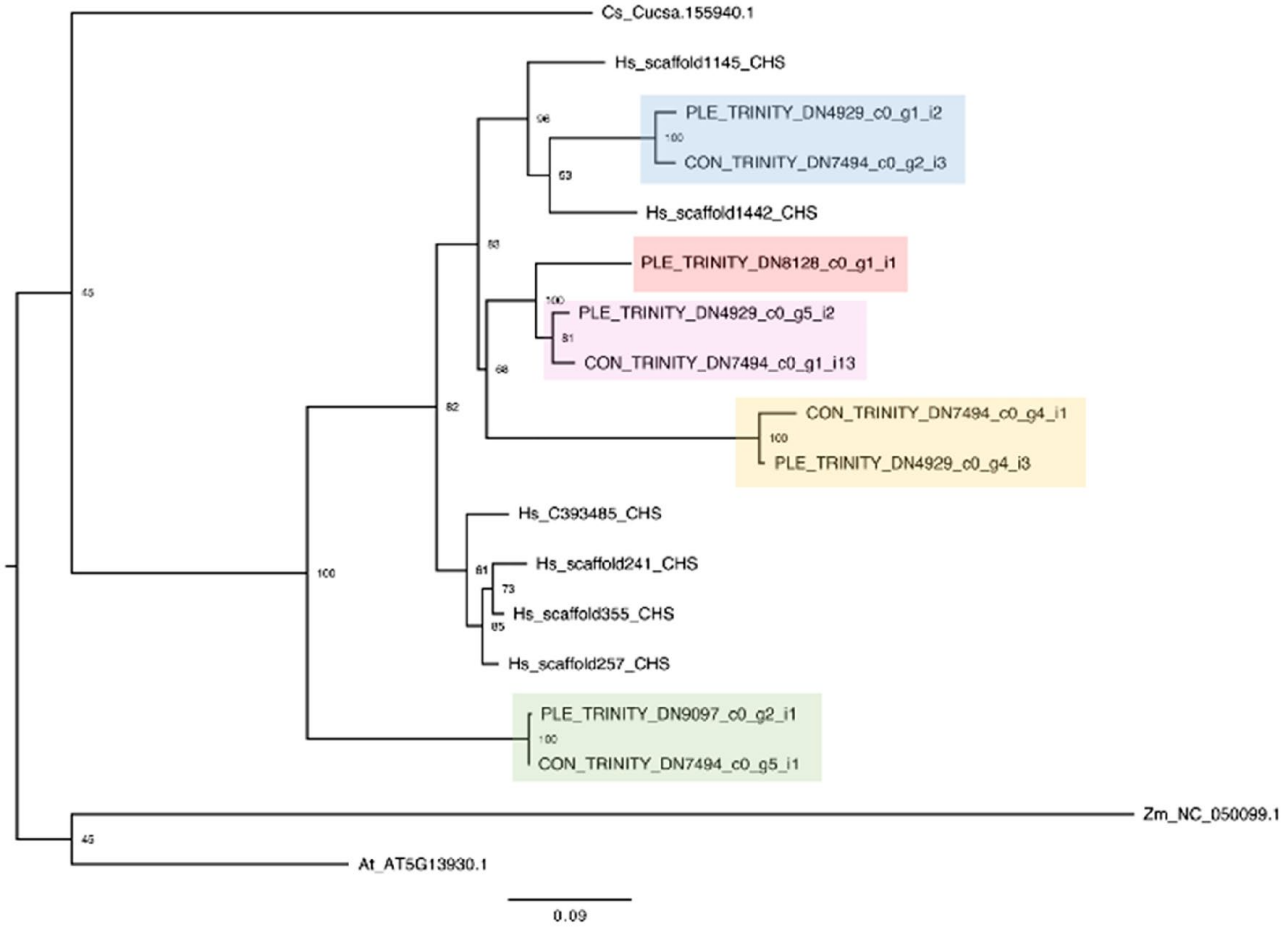
ML tree of CHS sequences from *B. conchifolia* (CON), *B. plebeja* (PLE), *H. sandwicensis* (Hs), *C. sativus* (Cs), *A. thaliana* (At) and *Z. mays* (Zm)*.* Pairs of orthologs are highlighted as follows: blue: group 1, pink; group 2, yellow: group 3, green: group 4, red: group 5.

**Figure 5:**
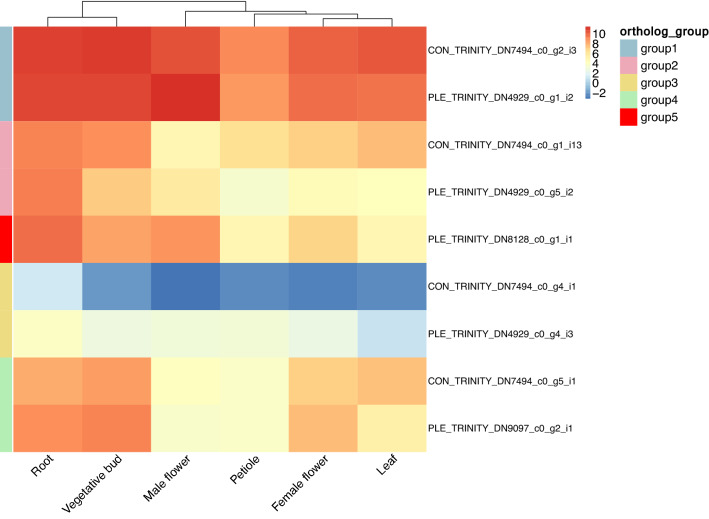
Heatmap of log2 FPKM of CHS homologs in *B. conchifolia* and *B. plebeja.* Ortholog groups refer to highlighted homologous CHS pairs in the ML gene tree.

The nine copies identified in *B. conchifolia* and *B. plebeja* are four putative orthologs and one single *B. plebeja* duplicate, and are colour coded for ease of comparison (Figs. 4 and 5).

The oldest duplication identified in *Begonia* gives rise to group 4 orthologs (in green, Fig. 4), and is placed after the divergence of *Begonia* and *Cucumis*.

The intra-ortholog expression similarity (e.g. group 1 *B. conchifolia* ortholog vs group 1 *B. plebeja* ortholog) is reasonably high (Fig. 5), and reflects the recent speciation of the two study species. The single *B. plebeja* ortholog in group 5 (red) also shows high similarity in expression profile to group 2 orthologs (pink), mirrored by the phylogenetic proximity of the two groups (Fig. 4). Any changes between each ortholog pair are therefore the result of expression changes since divergence of *B. conchifolia* and *B. plebeja*.

Inter-ortholog expression is more variable; ortholog group 1 (blue) has the highest expression (*B. conchifolia* FPKM min = 530, max = 2350, *B. plebeja* FPKM min = 408, max = 2903), and group 3 (yellow) has the lowest expression (*B. conchifolia* FPKM min = 0.11, max = 2.42, *B. plebeja* FPKM min = 2.06, max = 14.91). Ortholog groups 2 (*B. conchifolia* FPKM min = 27.57, max = 576.92, *B. plebeja* FPKM min = 11.91, max = 635.85 and 4 (*B. conchifolia* FPKM min = 13.65, max = 365.58, *B. plebeja* FPKM min = 12.26, max = 606.76) have much more comparable expression, indicating that phylogenetic proximity is not correlated with expression similarity in this case. The only exception is the group 5 single *B. plebeja* duplicate (FPKM min = 27.28, max = 871.03), which is the product of a duplication shortly before the group 2 orthologs, and shares similarity in expression due to phylogenetic proximity.

The group 3 CHS orthologs appear to have considerably decreased expression levels in both species, the *B. conchifolia* ortholog showing less than 1 FPKM expression across all tissues except for root.

**Figure 6:**
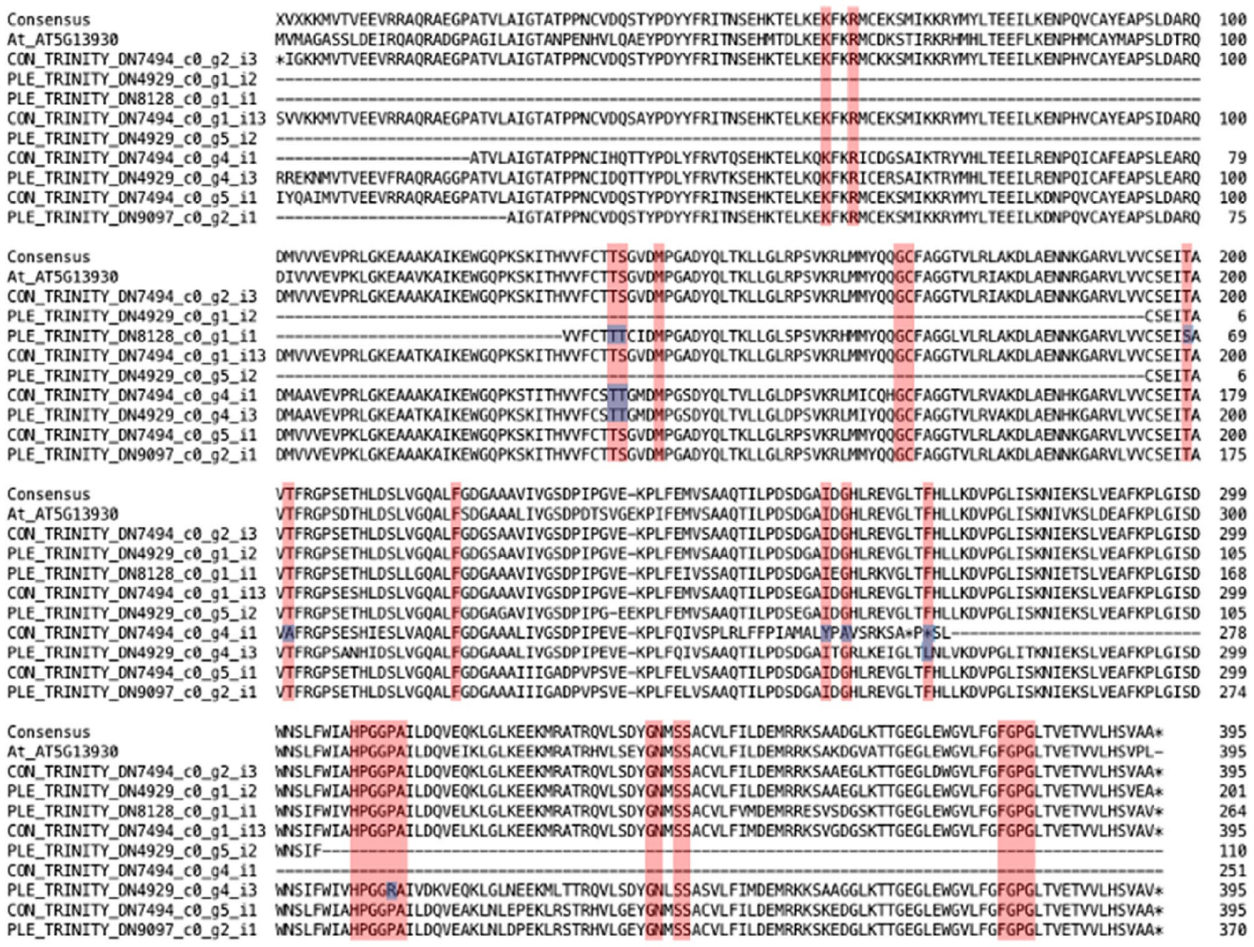
Multiple sequence alignment of CHS copies from *B. conchifolia*, *B. plebeja* and *A. thaliana*. Residues highlighted in red correspond to conserved sites obtained from the literature. Residues highlighted in blue are sites which differ in at least one *Begonia* sequence.

Examination of the peptide sequence alignment shows that the group 3 orthologs have a higher mutation rate, and the most peptide substitutions in conserved residues, some of which are catalytically important (Fig. 6). Moreover, examination of the coding sequence of the *B. conchifolia* group 3 ortholog shows that it has a frameshift mutation, resulting in two premature stop codons. While the *B. plebeja* group 3 ortholog also has mutations in conserved residues, it appears to have a complete peptide sequence. The only other CHS sequence which has a substitution at a conserved site is the single *B. plebeja* group 5 homolog, with three conserved sites showing a substitution.

Synonymous and nonsynonymous substitution rates were compared between ortholog pairs to estimate the level of selection acting on each pair of orthologs. A dN/dS ratio of < 1 indicates that the pair of sequences have more synonymous than nonsynonymous substitutions and are therefore under purifying selection. Conversely, a dN/dS ratio of > 1 indicates faster protein evolution, and that the sequences may be under positive selection.

All ortholog groups tested except for group 3 was under strong purifying selection (Table 4). Ortholog group 1 had the lowest dN/dS ratio, while group 4 had the most relaxed purifying selection. Group 3 orthologs showed a dN/dS ratio of 1.49, indicating relaxation of selective constraints on this pair of orthologs.

**Table 4:** Nonsynonymous substitutions (dN), synonymous substitutions (dS) and dN/dS ratio for ortholog pairs. Ortholog group refers to ortholog groupings in Figs. 4 and 5.

*B. conchifolia* ortholog	*B. plebeja* ortholog	Ortholog group	dN	dS	dN/dS
CON_TRINITY_DN7494_c0_g2_i3	PLE_TRINITY_DN4929_c0_g1_i2	Group 1	0.009	0.1064	0.0845
CON_TRINITY_DN7494_c0_g1_i13	PLE_TRINITY_DN4929_c0_g5_i2	Group 2	0.0118	0.1082	0.1090
CON_TRINITY_DN7494_c0_g4_i1	PLE_TRINITY_DN4929_c0_g4_i3	Group 3	0.0319	0.0213	1.4998
CON_TRINITY_DN7494_c0_g5_i1	PLE_TRINITY_DN9097_c0_g2_i1	Group 4	0.0012	0.0040	0.2953

## Discussion

The diversity of form seen across the Angiosperms is the topic of a wide scope of research, including conservation^41^, plant breeding^42^ and evolution^43^.

In this study, we address the role of gene and genome duplication in the generation of phenotypic and ecological diversity in *Begonia* using two closely related but ecologically distinct species, *B. conchifolia* and *B. plebeja*. We use multi-tissue RNA-seq to sample six tissues across both species, thereby also producing valuable transcriptomic sequence data to add to the growing genetic resources for *Begonia*.

We used contamination screening to find and remove contaminants. We identified a sizable number of sequences of non-plant origin representing environmental contamination during tissue collection and RNA extraction. Contamination of genomic and transcriptomic datasets is a widespread problem^44^, and while some contaminants are easy to spot, such as odorant binding proteins and chemosensory proteins unique to insects^45^, contaminants which are plausible homologs can pose a danger to the conclusions drawn from a study^46^. Our results have indicated that contaminants have lower coverage, presumably due to contaminant taxon tissue being more sparsely sampled, and therefore contribute to a large proportion of fragmented and incomplete transcripts. More transcript fragments may act to increase the perceived transcriptome complexity, and thus reduce the transcriptome assembly quality^47^.

After contaminant screening, 137,985,258 and 91,321,126 total reads were assembled into reference transcriptomes composed of 17,012 and 19,969 unigenes for *B. conchifolia* and *B. plebeja* respectively. Both transcriptomes had good metrics for completeness, 85.2% and 82.5% of BUSCOs were recovered completely in *B. conchifolia* and *B. plebeja* respectively and less than 10% of BUSCOs were missing in both species. Quality metrics suggest a higher rate of fragmented transcripts in *B. plebeja*; more total transcripts and a lower N50 and N90 (1905 and 842 and 2381 and 1090 for *B. conchifolia* and *B. plebeja* respectively).

More than 80% of both species’ transcriptomes were annotated using at least one source, providing valuable context to the reference transcriptomes as well as to tissue specific expression. Analysis of GO terms revealed leaves to be the most conserved in gene expression profiles between the two species and vegetative buds the most distinct. This may reflect the different developmental decisions during development of the leaf and meristems as the different leaf shapes and plant architectures are laid down, compared to very similar functional expression patterns in the mature leaf.

Multi-tissue RNA-seq allows for greater spatial resolution when investigating the fates of duplicated genes in isolation as well as within coexpression networks^48,49^. Expression divergence in gene duplicates is a key process that allows for tissue specialization and morphological diversification^50^, however these changes are also dependent on mode of duplication^50^. Further development of genomic data in *Begonia* may help to distinguish between the products of tandem and whole genome duplication and changes in their expression profiles since duplication.

Since the speciation of *B. conchifolia* and *B. plebeja*, their habitat range has diverged; the former lives in moist, shaded understorey, while the latter is found in open, seasonally dry forests^51^.

The different ecological niches occupied by our two study species may have driven their divergent phenotypic evolution, for example in their approach to light and water usage, and optimisation of photosynthetic capacity. In this study, we investigated the role duplicated genes may play in generating phenotypic and biochemical diversity.

We chose chalcone synthase (CHS), a key gene in the anthocyanin biosynthesis pathway, for further investigation due to the wide range of environmental responses it is involved in. While CHS is a key gene in the anthocyanin biosynthesis pathway with a role in attenuating high intensity light and acclimating to low light availability^52,53^, other roles of CHS include drought tolerance^54^ herbivory defense^55,56^, and defense against pathogens^57,58^.

Due to incomplete assembly of all CHS copies, four copies of CHS were investigated in *B. conchifolia* and five in *B. plebeja*, however both species have at least one additional copy of CHS that were excluded due to insufficient sequence length. CHS copies used in phylogenetic and expression analysis revealed they were all derived from duplications after the divergence of *Begonia* and *Cucumis*. The recent origin of all identified *Begonia* and *Hillebrandia* CHS copies may suggest a high turnover of the CHS gene family in the *Begoniaceae*; the loss of expression of old gene duplicates and a high duplication rate leading to only recent duplicates having a detectable expression level. The genomic source of CHS copies in *H. sandwicensis* supports a scenario of the loss of old CHS duplicates from the genome altogether, rather than retention in the genome with little to no detectable expression. Without a reference genome for our study species, it is not possible to say with certainty that this pattern is replicated in *Begonia.*

Of the five ortholog groups identified in CHS, three have detectable expression and an uninterrupted coding sequence in both species (groups 1, 2, and 4).

Group 3 orthologs have the lowest expression level, *B. conchifolia* not showing expression higher than 3 FPKM in any tissue, while *B. plebeja* has expression levels ranging from 2.1 to 14.9 FPKM. A frameshift mutation leading to two premature stop codons in the *B. conchifolia* group 3 ortholog is supportive of a loss of function mutation, resulting in this copy being non-functional in *B. conchifolia*. The *B. plebeja* group 3 ortholog does not show any obvious loss of function mutations, which may indicate that while its expression is low, it may still perform a function. The single member of ortholog group 5 is represented by *B. plebeja*, which showed a similar expression pattern to group 2, to which it was closest related, and well as group 4, possibly reflecting an ancestral expression pattern. The close proximity of the *B. plebeja* group 5 ortholog to the group 2 orthologs, and the absence of expression of a *B. conchifolia* ortholog suggests this may be a species specific duplication.

Examination of *Begonia* CHS coding sequences showed a variable level of synonymous and nonsynonymous substitutions across ortholog groups; the ortholog pair under the most relaxed evolutionary constraints was the very lowly expressed group 3 (Table 4), showing evidence of positive selection, concomitant with an apparent loss of function mutation in *B. conchifolia* and changes in the *B. plebeja* peptide sequence corresponding to conserved sites (Fig. 6). Increased expression in the *B. plebeja* group 3 ortholog relative to *B. conchifolia* may indicate that it still retains some function, and the positive selection acting on this CHS copy could allow the development of novel functionality.

The CHS gene family in *B. conchifolia* and *B. plebeja* has shown evidence of high duplicate turnover, as well as positive selection acting on some duplicate copies. High gene duplication rate is a prominent feature in taxa which have undergone phenotypic shifts, where new duplicate genes can be co-opted into a new developmental or biochemical program^59^. The asymmetric retention or duplication of genes, as seen in CHS in *Begonia* is an important mode of adaptation to new environments^60^ and identification of selectively retained duplicate genes can reveal functional biases of ecological importance^61^.

Adaptive evolution in combination with high duplicate turnover can allow the divergence of gene families between species through the rapid fixation of non-synonymous mutations^62^. New lineage specific duplicates may be preferentially retained and undergo adaptive evolution in response to environmental stresses^63^, and the open, high light environment of *B. plebeja* may exert selective pressure on genes related to optimising light harvesting, such as CHS.

Full data from a genome assembly is needed to resolve the likeliest evolutionary scenario, however the data presented here supports the hypothesis that chalcone synthase is highly dynamic in *Begonia* with signatures of rapid diversification at both the sequence and expression level and may be important in the phenotypic shifts that occurred after the speciation of *B. conchifolia* and *B. plebeja*.

## Conclusions

*Begonia* is a mega-diverse genus, with excellent applicability to research in conservation and plant breeding. We have produced a multi-tissue RNA-seq dataset in two closely related but morphologically and ecologically divergent species of *Begonia,* providing a valuable addition to the growing base of genomic resources in the genus. Recent duplications in an important anthocyanin biosynthetic gene, chalcone synthase, have led to sequence and expression divergence of duplicate copies, suggesting duplication patterns in this gene family are dynamic and prone to high turnover rates.

Further transcriptome wide investigations using the RNA-seq dataset generated here may help uncover species specific changes in expression profiles that have contributed to the ecological divergence of *B. conchifolia* and *B. plebeja* and answer wider questions about the role gene duplication plays in the generation of diversity in *Begonia*.

## Supplementary Information

Supplementary Information 1.

Supplementary Information 2.

Supplementary Information 3.

Supplementary Information 4.

Supplementary Information 5.
