# Supplementary material for: Multi-tissue transcriptome analysis of two Begonia species reveals dynamic patterns of evolution in the chalcone synthase gene family
Source: Sci Rep. 2021 Sep 7;11:17773. doi: 10.1038/s41598-021-96854-y (PMC8423730; doi:10.1038/s41598-021-96854-y)
Supplement: Supplementary file 1 — Supplementary Information 1. [file 41598_2021_96854_MOESM1_ESM.pdf]

Multi-tissue transcriptome analysis of two *Begonia* species reveals dynamic patterns of evolution in the chalcone synthase gene family

Katie Emelianova [1,2*], Andrea Martínez Martínez[1,3], Lucia Campos-Dominguez[1,3], Catherine Kidner [1,3]

[1] Royal Botanic Gardens Edinburgh, 20a Inverleith Row, Edinburgh, EH3 5LR UK

[2] Dementia Research Institute at the University of Edinburgh, Edinburgh, UK

[3] School of Biological Sciences, University of Edinburgh, King's Buildings, Mayfield Rd, Edinburgh, EH9 3JU UK

*Corresponding author

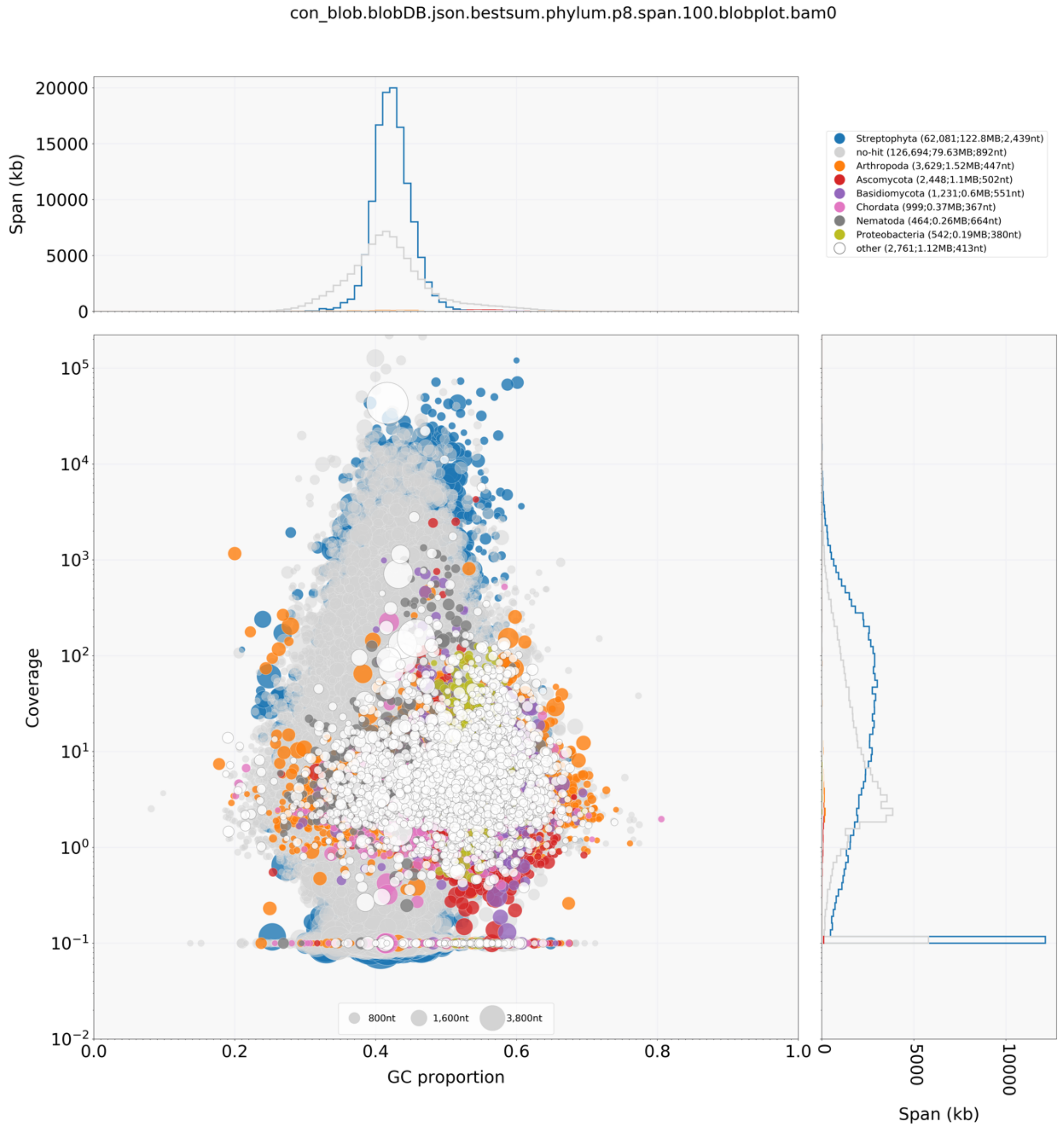

Supplementary figure 1. *B. conchifolia* BlobPlot showing taxonomic origin of transcripts

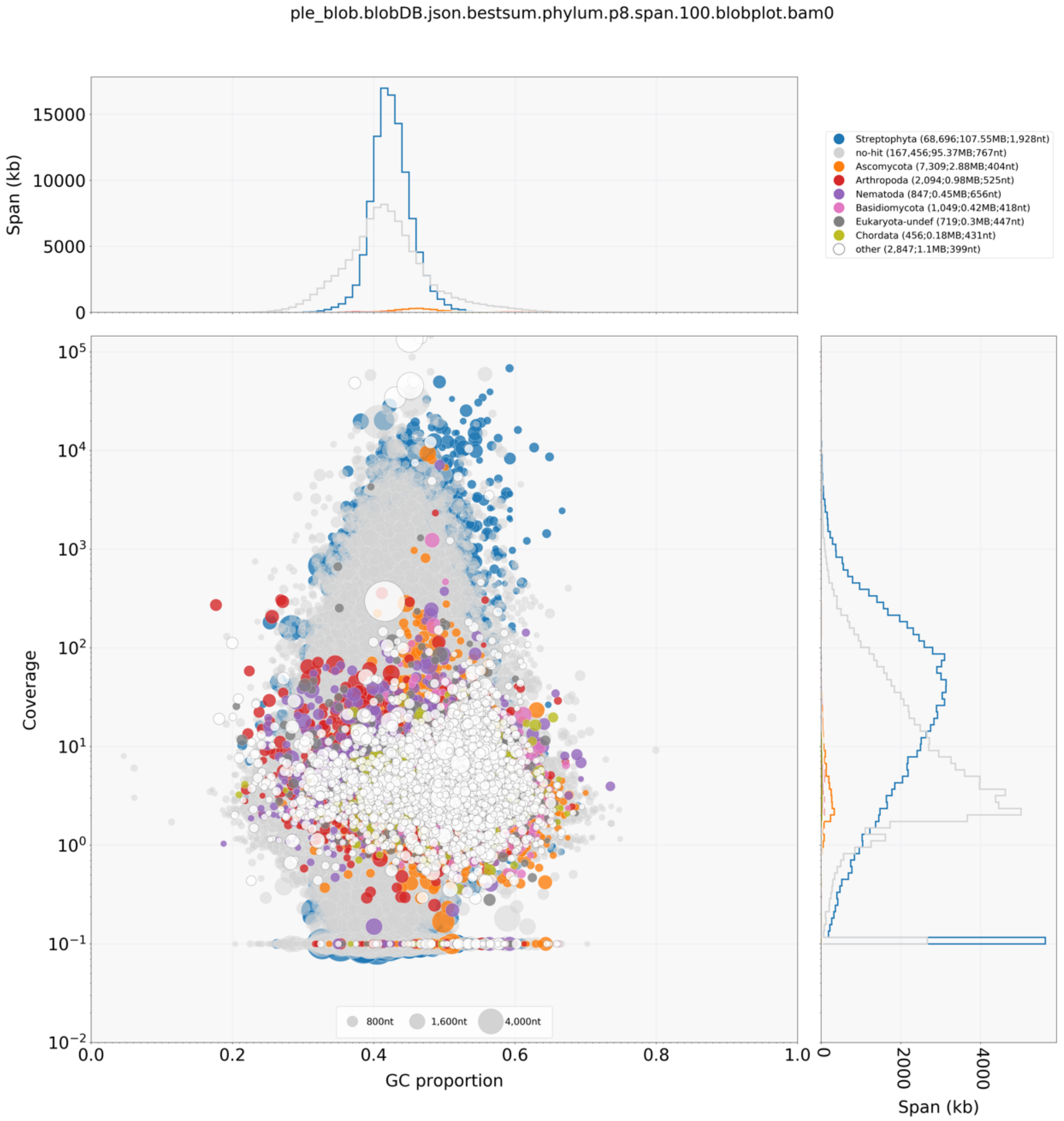

Supplementary figure 2. *B. plebeja* BlobPlot showing taxonomic origin of transcripts.

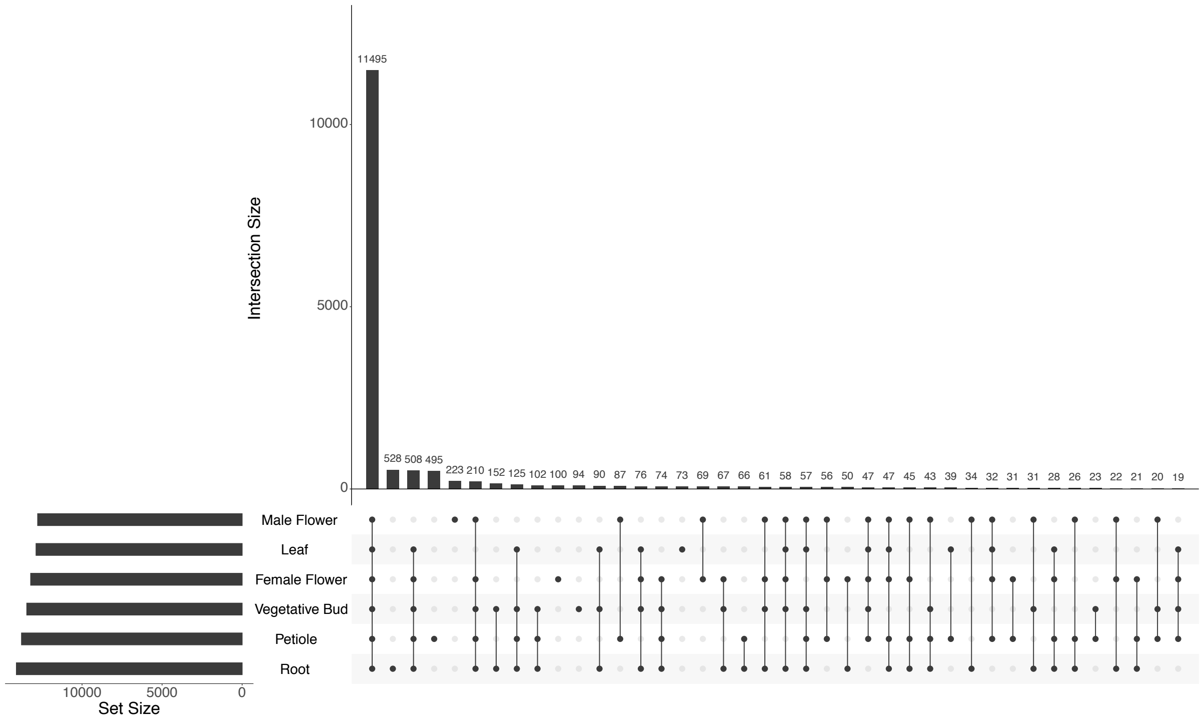

Supplementary figure 3. UpSet plot of number of transcripts shared between each tissue in *B. conchifolia*. Transcript presence/absence threshold was set at 1 FPKM.

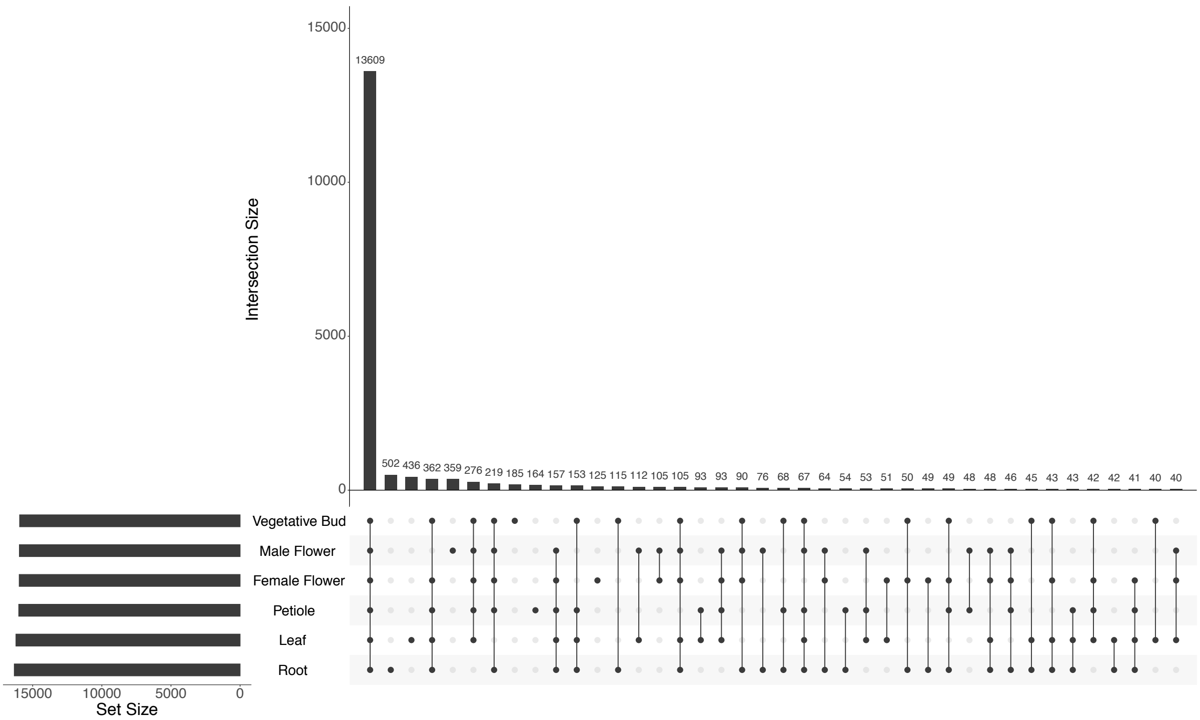

Supplementary figure 4. UpSet plot of number of transcripts shared between each tissue in *B. plebeja*. Transcript presence/absence threshold was set at 1 FPKM.

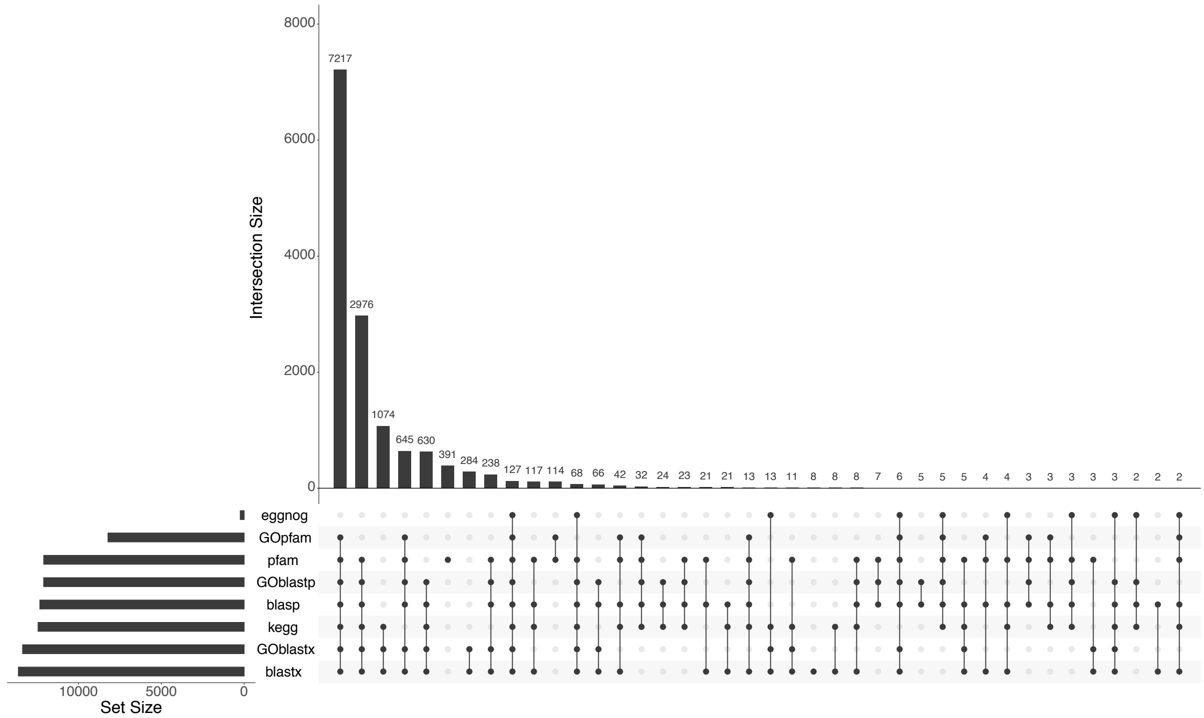

Supplementary figure 5. UpSet plot of sources of annotation of *B. conchifolia* unigenes.

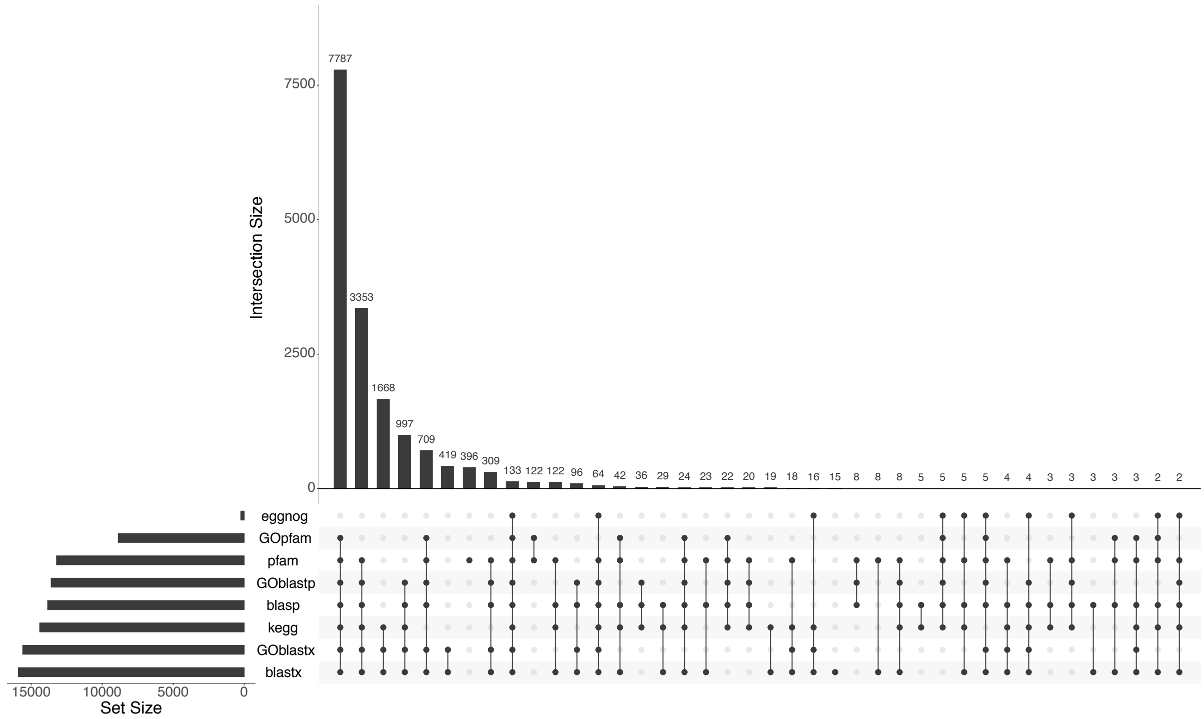

Supplementary figure 6. UpSet plot of sources of annotation of *B. plebeja* unigenes.

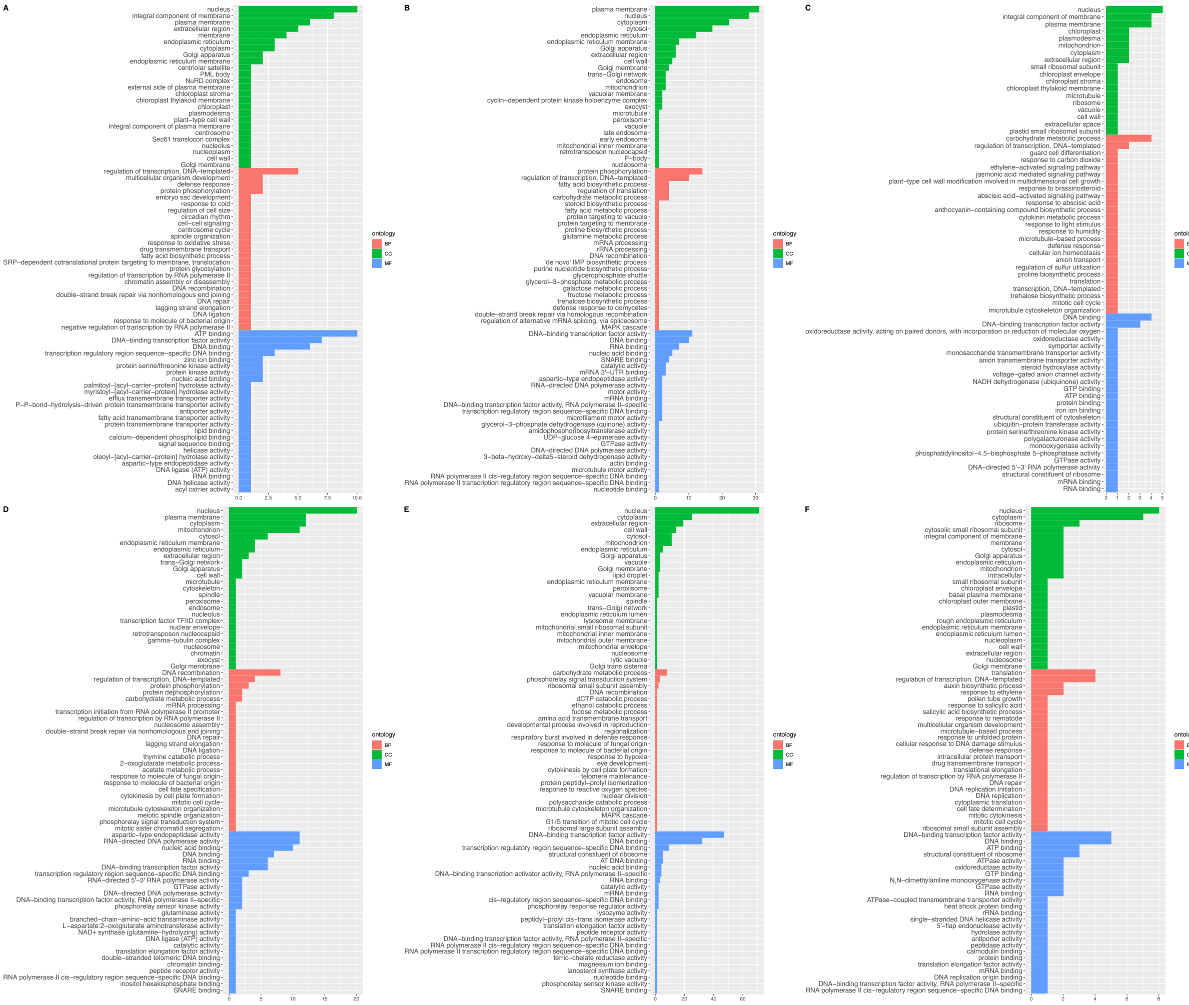

Supplementary figure 7. GO terms mapped to transcripts which are uniquely expressed in *B. conchifolia* tissues (A: female flower, B: male flower, C: leaf, D: petiole, E: root, F: vegetative bud).

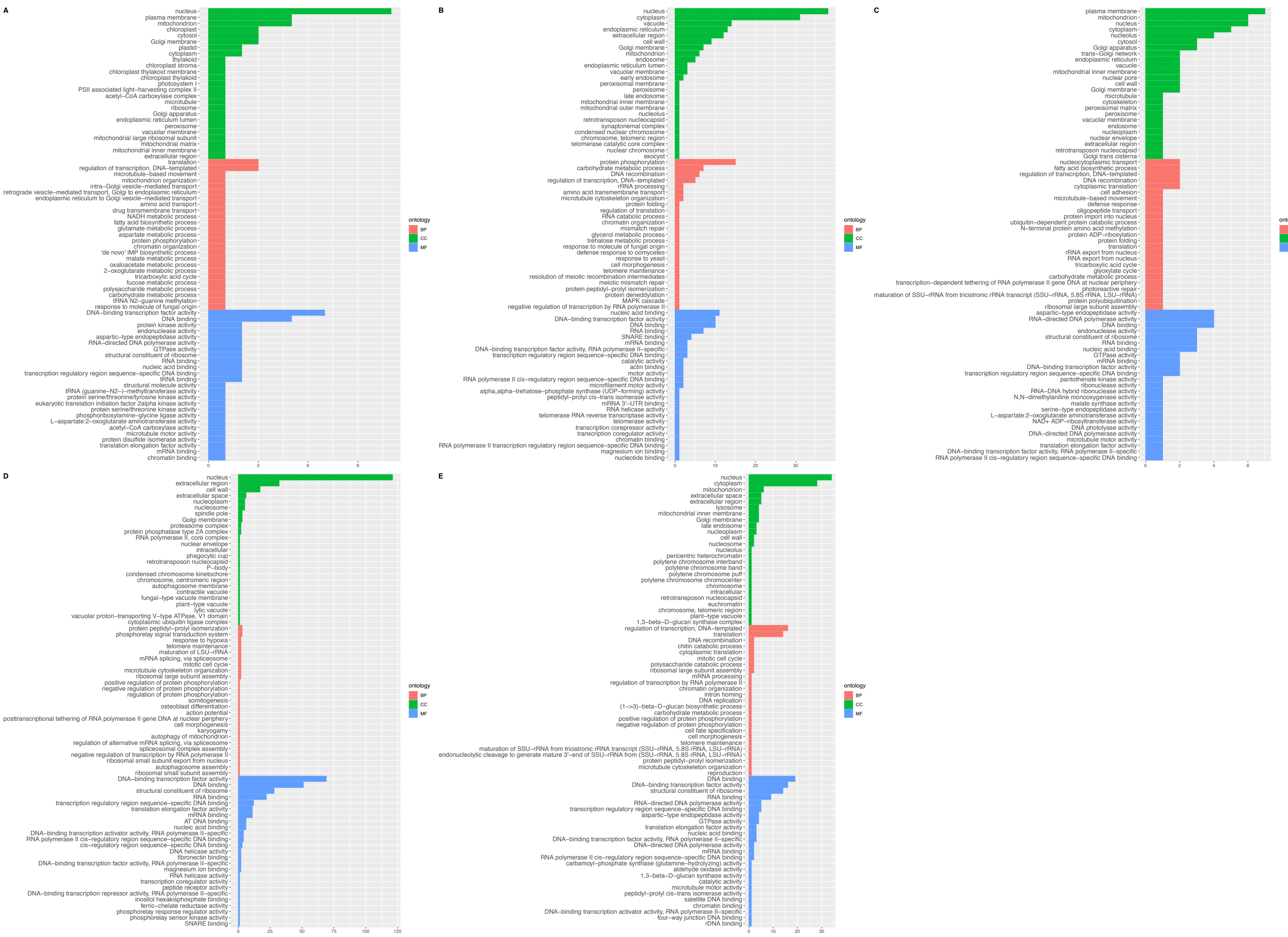

Supplementary figure 8. GO terms mapped to transcripts which are uniquely expressed in *B. plebeja* tissues (A: female flower, B: male flower, C: leaf, D: root, E: vegetative bud).

	B. conchifolia before BlobTools	*B. plebeja* before BlobTools	*B. conchifolia* after BlobTools	*B. plebeja* after BlobTools
Female flower 1	9,891,456	10,032,200	6,854,350	6,286,106
Female flower2	10,617,146	7,385,354	7,112,514	4,571,012
Female flower3	8,015,202	8,117,202	5,077,862	4,838,732
Leaf1	8,870,430	9,471,050	5,703,088	6,129,172
Leaf2	6,686,284	8,521,928	4,577,202	5,609,244
Leaf3	7,693,508	9,725,984	5,074,194	6,555,628
Male flower1	11,531,146	10,118,246	6,847,048	5,634,006
Male flower2	9,836,190	12,980,896	5,615,906	7,475,420
Male flower3	67,859,634	10,572,966	39,271,692	6,197,852
Petiole1	8,946,782	9,234,410	5,831,818	5,385,578
Petiole2	8,969,440	11,087,144	6,035,118	6,414,900
Petiole3	6,945,578	NA	4,432,814	NA
Root1	9,038,560	7,321,210	5,936,038	4,050,034
Root2	NA	6,465,758	NA	3,572,410
Root3	10,652,432	7,863,298	7,337,244	3,840,676
Veg bud1	11,837,738	9,424,268	8,132,896	5,008,742
Veg bud2	13,109,894	8,899,078	9,035,192	5,153,244
Veg bud3	7,551,984	8,596,202	5,110,282	4,598,370
Total	218,053,404	155,817,194	137,985,258	91,321,126

Supplementary table 1. Read counts before and after contaminant removal for *B. conchifolia* and *B. plebeja*. NA values indicate libraries that had to be discarded due to technical issues.

Species	Sequence ID	% Identity	E value	Sequence length
B. conchifolia	TRINITY_DN7494_c0_g1_i13	86.5	0.0	1544
B. conchifolia	TRINITY_DN7494_c0_g5_i1	86.010	0.0	1867
B. conchifolia	TRINITY_DN7494_c0_g2_i3	79.717	0.0	1777
B. conchifolia	TRINITY_DN7494_c0_g4_i1	70.815	3.08e-122	753
B. conchifolia	TRINITY_DN7494_c0_g3_i1	79.200	3.11e-67	381
B. plebeja	TRINITY_DN9097_c0_g2_i1	87.500	0.0	1252
B. plebeja	TRINITY_DN4929_c0_g4_i3	76.166	0.0	2406
B. plebeja	TRINITY_DN8128_c0_g1_i1	85.878	7.73e-165	1027
B. plebeja	TRINITY_DN4929_c0_g1_i2	90.955	2.01e-126	772
B. plebeja	TRINITY_DN9097_c0_g1_i2	81.212	4.22e-99	695
B. plebeja	TRINITY_DN4929_c0_g5_i2	87.387	3.84e-61	332
B. plebeja	TRINITY_DN5410_c0_g2_i4	69.369	1.07e-52	458
B. plebeja	TRINITY_DN9097_c0_g3_i1	88.095	1.47e-43	251
B. plebeja	TRINITY_DN5410_c0_g4_i1	71.429	1.42e-27	249
B. plebeja	TRINITY_DN5410_c0_g5_i1	71.429	3.76e-27	206
B. plebeja	TRINITY_DN5410_c0_g1_i3	70.149	9.84e-26	1944

Supplementary table 2. Results of tblastn searches of an *A. thaliana* copy of CHS against *B. conchifolia* and *B. plebeja*
